# Incipient Fault Detection for Rolling Element Bearings under Varying Speed Conditions

**DOI:** 10.3390/ma10060675

**Published:** 2017-06-20

**Authors:** Lang Xue, Naipeng Li, Yaguo Lei, Ningbo Li

**Affiliations:** Key Laboratory of Education Ministry for Modern Design and Rotor-Bearing System, School of Mechanical Engineering, Xi’an Jiaotong University, Xi’an 710049, China; 18732195574@163.com (L.X.); li3112001096@stu.xjtu.edu.cn (N.L.); liningbo1992@163.com (N.L.)

**Keywords:** incipient fault detection, varying speed, alarm trigger mechanism, adaptive threshold, rolling element bearings

## Abstract

Varying speed conditions bring a huge challenge to incipient fault detection of rolling element bearings because both the change of speed and faults could lead to the amplitude fluctuation of vibration signals. Effective detection methods need to be developed to eliminate the influence of speed variation. This paper proposes an incipient fault detection method for bearings under varying speed conditions. Firstly, relative residual (RR) features are extracted, which are insensitive to the varying speed conditions and are able to reflect the degradation trend of bearings. Then, a health indicator named selected negative log-likelihood probability (SNLLP) is constructed to fuse a feature set including RR features and non-dimensional features. Finally, based on the constructed SNLLP health indicator, a novel alarm trigger mechanism is designed to detect the incipient fault. The proposed method is demonstrated using vibration signals from bearing tests and industrial wind turbines. The results verify the effectiveness of the proposed method for incipient fault detection of rolling element bearings under varying speed conditions.

## 1. Introduction

Rolling element bearings play a vital role in various industrial fields such as aviation, energy, and metallurgy. They generally work under varying speed conditions, which brings great challenges to the health monitoring and performance degradation evaluation of bearings [1]. For instance, the rotating speed of wind turbines changes because of time-varying wind speed and grid control over the long term. In such operation conditions, vibration signals always show non-stationary characteristics. Both amplitudes and frequency components of vibration signals change with time [2,3]. Traditional health monitoring methods generally assume that the change of vibration signals is just caused by faults of mechanical equipment [4]. Therefore, these methods are unsuitable to health monitoring in the varying speed conditions. Aiming for the issue of incipient fault detection of rolling element bearings under varying speed conditions, new health monitoring methods should be developed. Basically, there are two crucial factors determining the accuracy of incipient fault detection under varying speed conditions: health indicator extraction and alarm trigger mechanism. 

In the incipient fault detection process, a health indicator is always desired to effectively compress the large amount of vibration signals and reflect the degradation trend of rotating machinery. Many studies have been conducted on constructing such health indicators. Niu et al. [5] exploited self-organizing map neural networks to extract a minimum quantization error fusion indicator for methane compressor health monitoring. Soualhi et al. [6] used the Hilbert–Huang transform to extract Hilbert spectral density as a health indicator to track the degradation of the critical components of bearings. Li et al. [7] extracted a compressed acoustic emission indicator based on the empirical model decomposition for gear fault detection. Guo et al. [8] proposed a recurrent neural network based health indicator for remaining useful life prediction of bearings. Javed et al. [9] extracted a novel indicator based on trigonometric functions and cumulative transformation to monitor the degradation trend of cutting tools and bearings. Qian et al. [10] applied recurrence quantification analysis to extract recurrence plot entropy indicators for monitoring bearing performance degradation. Picot et al. [11] proposed a statistic-based spectral health indicator to detect bearing faults. These indicators are able to reflect the degradation trend of rotating machinery, but they are hard to avoid being influenced by the operation condition change. On the contrary, indicators such as kurtosis are insensitive to the change of speed condition, but they are unable to reflect the degradation trend of rotating machinery. Generally speaking, a desirable monitoring indicator should predominantly satisfy the following two requirements: (1) being insensitive to the change of operation conditions; and (2) reflecting the degradation degree of fault severity quantitatively. Considering the shortcoming of existing indicators, it is necessary to extract new health indicators for the incipient fault detection under varying speed conditions.

After constructing health indicators, an alarm trigger mechanism is needed to judge whether incipient faults occur on rotating machinery or not. Various fault trigger mechanisms have been designed to deal with this problem. Ginart et al. [12] presented a fault trigger mechanism based on constant alarm threshold for fault detection of rotating machinery. Li et al. [7] detected gearbox faults using a probability trigger mechanism, i.e., if more than a given percentage of features are greater than a fixed fault detection threshold, then it is believed that the incipient fault happens. Loutas et al. [13] employed an alarm trigger mechanism based on a heuristic threshold for health monitoring of gearboxes. Li et al. [14] proposed an adaptive continuous trigger mechanism, which is regarded that the incipient fault has occurred when several continuous indicator values exceed the 3σ interval. Jin et al. [15] employed a combined alarm trigger mechanism to successfully detect faults of cooling fans and motor bearings. These approaches have good performance in the fault detection. However, they still have several shortcomings. For instance, in the probability trigger mechanism, the percentage is calculated based on all of the historical measurements, which changes little when a new indicator value above the threshold appears. In the continuous trigger mechanism, several continuous indicator values above the threshold often occur in the significant faults stage, but seldom happen in the incipient fault stage.

To deal with the aforementioned problems, this paper proposes an incipient fault detection method for rolling element bearings under varying speed conditions. The major contributions of this method are: (1) relative residual (RR) features based on extreme learning machine (ELM) are proposed, which are insensitive to the varying speed conditions and are able to better reflect the degradation trend of bearings; (2) a health indicator, i.e., selected negative log-likelihood probability (SNLLP), is constructed to fuse multiple features; and (3) an alarm trigger mechanism is established to improve the accuracy of incipient fault detection based on the analysis of the fault typical behavior.

The rest of this paper is organized as follows. Section 2 describes the basic theory of ELM and Gaussian mixture model (GMM), respectively. In Section 3, the proposed incipient fault detection method is introduced in detail. In Section 4, the health state of the industrial wind turbine is monitored to further verify the effectiveness of the proposed method. Some concluding remarks are drawn in Section 5.

## 2. Brief Introduction to ELM and GMM

### 2.1. Extreme Learning Machine

ELM was proposed by Huang for single hidden layer feedforward neural networks (SLFNs) [16]. The learning speed of the feedforward neural network is constrained by gradient-based iterative learning algorithms. Unlike many other popular learning algorithms, the learning speed of ELM is fast because input weights and hidden neuron biases are randomly generated [17]. ELM has been widely used for regression and classification issues by virtue of few parameters tuning, fast learning speed and good generalization performance.

**Figure 1:**
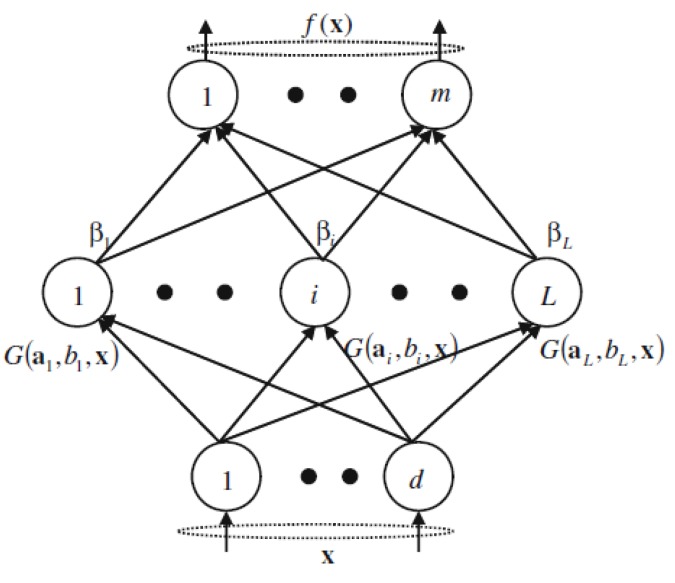
The architecture of SLFNs.

The output of SLFNs with *L* hidden nodes can be expressed as:

(1) $$f_{L}\left(x\right)=\overset{L}{\underset{i=1}{\sum }}\beta_{i}G\left(a_{i},b_{i},x\right),$$

where $a_{i}$ is the weight vector connecting input nodes and the *i*th hidden node, $b_{i}$ is the bias of the *i*th hidden node, $\beta_{i}$ is the weight connecting the *i*th hidden node to the output node and $G\left(a_{i},b_{i},x\right)$ is the output of the *i*th hidden node with respect to the input *x*. The architecture of SLFNs is shown in the Figure 1.

The N samples ${\{\left(x_{i},\;t_{i}\right)\}}_{i=1}^{N}$ are used to train parameters of SLFNs, where $x_{i}$ is an n×1 input vector and $t_{i}$ is a m×1 target vector.

Then, Equation (1) can be rewritten as

(2) Hβ=T,

where

(3) $$H=\left[\begin{array}{c} h\left(x_{1}\right)\\ \vdots \\ h\left(x_{N}\right) \end{array}\right]={\left[\begin{array}{ccc} G\left(a_{1},b_{1},x_{1}\right) & \ldots & G\left(a_{L},b_{L},x_{1}\right)\\ \vdots & \ldots & \vdots \\ G\left(a_{1},b_{1},x_{N}\right) & \ldots & G\left(a_{L},b_{L},x_{N}\right) \end{array}\right]}_{N\times L},$$

(4) $$\beta ={\left[\begin{array}{c} \beta_{1}^{T}\\ \vdots \\ \beta_{L}^{T} \end{array}\right]}_{L\times m}\;\mathrm{and}\;T={\left[\begin{array}{c} t_{1}^{T}\\ \vdots \\ t_{L}^{T} \end{array}\right]}_{N\times m},$$

The number of hidden nodes L is always less than the number of training samples, which means that the training error cannot be exactly zero but can approach zero. ELM randomly generates the input weight $a_{i}$ and the hidden node bias $b_{i}$, so the output weight $\beta_{i}$ is just needed to be solved, which is estimated as

(5) $$\hat{\beta }=H^{+}T,$$

where $H^{+}$ is a Moore–Penrose generalized inverse of the H matrix.

For a given training sample set ${\{\left(x_{i},\;t_{i}\right)\}}_{i=1}^{N}$ and *L* hidden nodes, the solution process of ELM parameters is listed as:

Step 1: Randomly generate the hidden layer parameters $\left(a_{i},b_{i}\right),$ i=1,…,L.

Step 2: Select an infinitely differentiable function as the activation function for hidden nodes and then calculate the output matrix of hidden layer H.

Step 3: Calculate the output weight $\beta :\hat{\beta }=H^{+}T$. 

### 2.2. Gaussian Mixture Model

GMM has strong robustness and excellent computing performance to model the complicated internal distribution characteristic contained in the data by using several Gaussian mixture components [18]. This characteristic makes it very suitable to smooth the probability distribution of arbitrary shape. Therefore, the GMM has been widely used in image background extraction and speech recognition in recent years [19].

Theoretically, arbitrary data can be expressed by *M* Gaussian probability density functions

(6) $$p(x_{i}|\Phi )=\overset{M}{\underset{j=1}{\sum }}\alpha_{j}N_{j}\left(x_{i},\mu_{j},{\sum^{\text{​}}}_{j}\right),$$

where $\alpha_{j}$ is the weight of the *j*th mixture component that satisfies the constraint $\overset{M}{\underset{j=1}{\sum }}\alpha_{j}=1$ and $0\leq \alpha_{j}\leq 1$, j=1,2,…,M. $N_{j}\left(x_{i},u_{j},{\sum^{\text{​}}}_{j}\right)$ is a unimodal Gaussian probability density function, which is expressed as

(7) $$N\left(x;u,\sum^{\text{​}}\right)=\frac{1}{\sqrt{{\left(2\pi \right)}^{n}\left|\sum^{\text{​}}\right|}}\exp\left[-\frac{1}{2}{\left(x-u\right)}^{T}{\sum^{\text{​}}}^{-1}\left(x-u\right)\right],$$

where x is the *n*-dimensional data vector, u is a n×1 mean vector and ∑ is a n×n covariance matrix.

To a N×D dimensional training sample set $X={\left\{x_{1},x_{2},\ldots ,x_{N}\right\}}^{T},$ the sample set is used to estimate the GMM parameters $\varphi_{j}=\left(x_{i},u_{j},{\sum^{\text{​}}}_{j}\right)$ with the aid of the expectation–maximization (EM) algorithm. The solution process of the GMM parameters is listed as follows:

Step 1: The *K*-means clustering algorithm is first utilized to initialize GMM parameters $x_{j0},u_{j0},{\sum^{\text{​}}}_{j0}$.

Step 2: To each training data $x_{i}$, estimate the probability produced by each unimodal Gaussian probability density function. The probability produced by the *j*th Gaussian mixture component is expressed as

(8) $$\beta_{ij}=\frac{\alpha_{j}N_{j}\left(x_{i};\Phi \right)}{\sum_{k=1}^{M}\alpha_{k}N_{k}\left(x_{i};\Phi \right)},1\leq i\leq n,1\leq j\leq M;$$

Step 3: Update parameters $x_{j},u_{j},{\sum^{\text{​}}}_{j}$.

(9) $$\alpha_{j}=\frac{\sum_{i=1}^{N}\beta_{ij}}{N},u_{j}=\frac{\sum_{i=1}^{N}x_{i}\beta_{ij}}{\sum_{i=1}^{N}\beta_{ij}},{\sum^{\text{​}}}_{j}=\frac{\sum_{i=1}^{N}\beta_{ij}\left(x_{i}-{u_{j}}^{T}\right){\left(x_{i}-{u_{j}}^{T}\right)}^{T}}{\sum_{i=1}^{N}\beta_{ij}};$$

Step 4: Repeatedly iterate Steps 2 and 3 to update the parameters $x_{j},u_{j},{\sum^{\text{​}}}_{j}$ until $\left|p{\left(X|\Phi \right)}^{k+1}-p{\left(X|\Phi \right)}^{k}\right|<\varepsilon$, where $p{\left(X|\Phi \right)}^{k}$ and $p{\left(X|\Phi \right)}^{k+1}$ are the probability density of *k*th and (*k*+1)th iteration, respectively.

Once the final parameters are determined, the GMM that is trained by the sample set has been constructed. Then, the trained GMM can be used to evaluate the membership degree of new data relative to the training sample set, which is able to be denoted by the $p(x_{i}|\Phi )$.

## 3. Proposed Incipient Fault Detection Method

**Figure 2:**
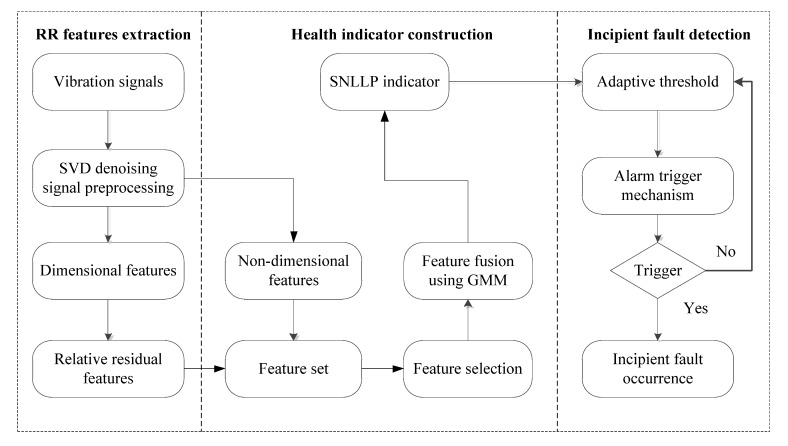
Flowchart of the proposed incipient fault detection method.

The flowchart of the proposed method is shown in Figure 2. It is composed of three modules, i.e., RR extraction, health indicator construction and incipient fault detection.

In the RR features extraction module, real-time vibration signals acquired from rotating machinery are de-noised by singular value decomposition (SVD) to enhance weak incipient fault characteristics. Then, dimensional features are extracted from the de-noised signals. Since the dimensional features are easily influenced by speed variation, RR features are proposed to reduce the interference and better reflect the health degradation trend of bearings. In the health indicator construction module, a feature set is made up of RR features and non-dimensional features. Then, some features are selected from the feature set using degradation evaluation metrics, and the dimension of these selected features is further reduced by orthogonal locality preserving projections (OLPP). A novel health indicator called SNLLP is constructed based on GMM to comprehensively quantify performance degradation of bearings. In the incipient fault detection module, based on the SNLLP indicator, the incipient fault is detected by the proposed alarm trigger mechanism. More details about these three modules are presented as follows.

### 3.1. RR Features Extraction

In the RR features extraction module, at first, real-time vibration signals are acquired from sensors attached on rolling element bearings. Since bearing fault characteristics, especially in the incipient stage, are always submerged in strong background noise, SVD is employed to cancel the noise imbedded in vibration signals [20]. Then, original features are extracted from the de-noised signals in time domain, frequency domain and time-frequency domain. Generally, these features are classified into two classes, i.e., dimensional features and non-dimensional features. Many dimensional features are always able to directly reflect the health degradation trend of bearings at a constant speed condition, but the amplitudes of these features are also susceptible to varying speed conditions. To explore the relationships between the speed and dimensional features, varying speed experiments of bearings are executed in a test rig. 

**Figure 3:**
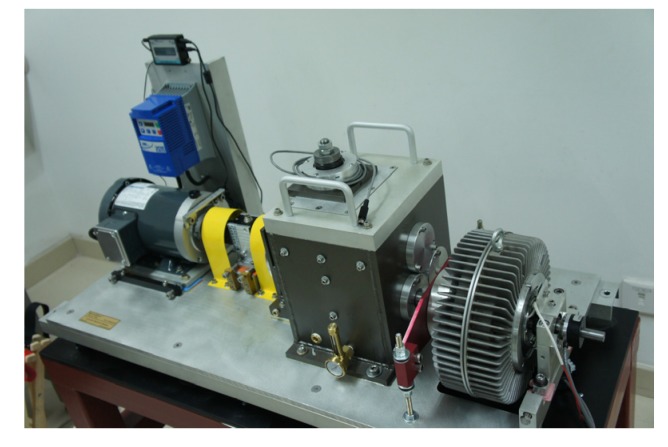
A bearing test rig.

**Figure 4:**
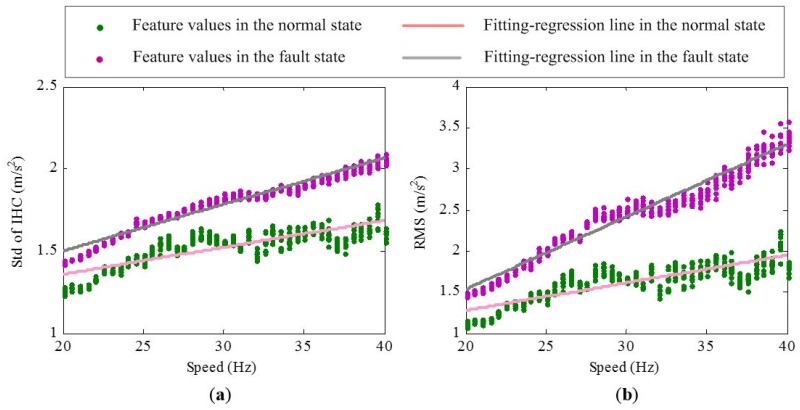
Variation of feature values with the increase of speed under normal and fault states: (**a**) Std of IHC; (**b**) RMS.

The experimental system is shown in Figure 3. The test rig includes two bearings, a 3-hp motor for driving bearing operation. The motor rotating speed is controlled by a speed controller, which allows the tested bearing to operate under various speeds. Accelerometers are fixed on end cap closing to the tested bearing. The sampling frequency is 25.6 kHz. Data acquisition equipment is utilized to collect the vibration signals of bearings in normal and fault states via increasing the motor speed from 20 Hz to 40 Hz. The speed keeps stable for a period after increasing each 0.5 Hz. Under each constant-speed period, 10 sets of vibration signals are acquired. Each set of vibration signal contains 76,800 data points, i.e., 3 s. Figure 4 shows the variation of standard deviation of inverse hyperbolic cosine (Std of IHC) [9] and root mean square(RMS) values with the increase of speed under normal and fault states. From Figure 4, two obvious rules could be concluded:

- There is an approximately linear relationship between the motor speed and the extracted features whether in the normal state or fault state.
- The fitting-regression lines of the features under the fault state have a larger slope than those under a normal state, which means that the bearing in the fault state is more susceptible to speed than the bearing in the normal state.

**Figure 5:**
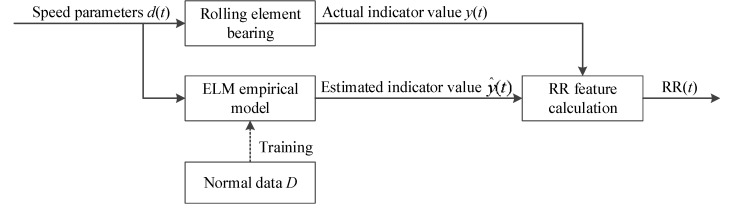
Schematic of RR feature extraction.

In order to reduce the influence of the time-varying speed to dimensional features, the dimensional features are transformed to a set of RR features using ELM to reflect the degradation trend of rotating machinery under varying speed conditions. The process of RR feature extraction is shown in Figure 5.

Step 1: The speed condition parameters in the normal state $d_{t}\in R^{B\times 1}$ is used as the input of the trained model and the dimensional feature in the normal state $F_{t}\in R^{B\times 1}$ is utilized as the output of the trained model to train the ELM empirical model of the normal behavior, where *B* is the number of training samples.

Step 2: The new captured operating speed d(t) is used as the input of the trained model. The ELM model estimation output value of d(t) can be obtained as $\hat{y}=f\left(d\right)$.

Step 3: The RR feature is constructed as $RR=\left(y-\hat{y}\right)/\hat{y}$, where y is the actual feature value extracted from the measured vibration signal.

Feature values in the fault state will deviate from the distribution of those in the normal state, leading to statistically abnormal changes in RR values. Therefore, faults can be detected by evaluating the extracted RR values.

**Figure 6:**
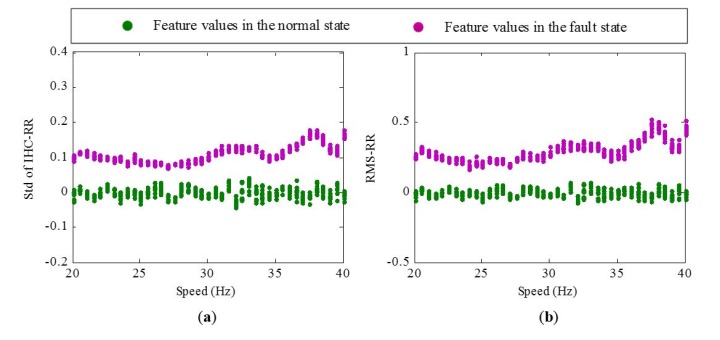
Results of the extracted RR features: (**a**) Std of IHC-RR; (**b**) RMS-RR.

To evaluate the effectiveness of the proposed RR features, experimental data are further analyzed. As described above, vibration signals of bearings in normal and fault states within the 20–40 Hz speed range are first de-noised by the SVD. Then, Std of IHC and RMS are selected from the dimensional features to demonstrate the performance of corresponding RR features. The Std of IHC and RMS values in the normal state and corresponding speed parameters are used to train the ELM empirical model. The performance of the extracted RR features feature in the normal and fault state is shown in Figure 6. It is seen that the amplitudes of RR features in the normal state are close to 0 and stay stable. In the same fault degree, the amplitudes of RR features are greater than 0. In addition, they are still stable without being influenced by the speed variation obviously. The results indicate that RR features can well recognize the normal and fault state of bearings and also have a good resistance to speed variation.

### 3.2. Health Indicator Construction

In this module, GMM-based SNLLP is constructed to provide a fusion health indicator. When the feature set composed of RR features and non-dimensional features is extracted, an optimal degradation feature selection method is required to select the features with useful information about bearing degradation processes. Good condition monitoring features should be well correlated to bearing degradation progresses, monotonous increasing or decreasing, robustness to outliers caused by noise, etc. Therefore, correlation, monotonicity and robustness metrics are utilized here for optimal feature selection. An integrated metric about these three evaluation metrics is used [21]:

(10) $$J=w_{1}Corr\left(F\right)+w_{2}Mon\left(F\right)+w_{3}Rob\left(F\right),$$

(11) $$s.t.\{\begin{array}{c} w_{i}>0\\ \underset{i}{\sum }w_{i}=1,i=1,2,3 \end{array},$$

where J is total degradation evaluation score whose values belong to the interval [0,1], Corr(F,T) is the correlation metric, Mon(F) is the monotonicity metric, Rob(F) is the robustness metric and $w_{i}$ is the weight of each metric. Three degradation evaluation metrics are computed by virtue of the following equations:

(12) $$Corr\left(F,T\right)=\frac{\left|K\sum_{k}F_{T}\left(t_{k}\right)t_{k}-\sum_{k}F_{T}\left(t_{k}\right)t_{k}\right|}{\sqrt{\left[K\sum_{k}F_{T}{\left(t_{k}\right)}^{2}-{\left(\sum_{k}F_{T}\left(t_{k}\right)\right)}^{2}\right]\left[K\sum_{k}{t_{k}}^{2}-{\left(\sum_{k}t_{k}\right)}^{2}\right]}},$$

(13) $$Mon\left(F\right)=\frac{1}{K-1}\left|\underset{k}{\sum }\delta \left(F_{T}\left(t_{k+1}\right)-F_{T}\left(t_{k}\right)\right)-\underset{k}{\sum }\delta \left(F_{T}\left(t_{k}\right)-F_{T}\left(t_{k+1}\right)\right)\right|,$$

(14) $$Rob\left(F\right)=\frac{1}{K}\underset{k}{\sum }\exp\left(-\left|\frac{F_{R}\left(t_{k}\right)}{F\left(t_{k}\right)}\right|\right),$$

where $F\left(t_{k}\right)$ is the degradation feature value at the time $t_{k}$, $F_{T}\left(t_{k}\right)$ is the trend value which can be obtained by common smoothness method, $F_{R}\left(t_{k}\right)$ is the residual random value, K is the number of total feature values and δ(⋅) is the unit step function.

In order to select the degradation features that have good performance, the features whose J have higher scores are primarily selected. The selected degradation features still have redundant information. Therefore, the dimension of these selected features is further reduced by OLPP. The OLPP is utilized here to retain the low-dimensional nonlinear manifold structure that is hidden in the data set and better dig out intrinsic fault information [22]. The former principal components whose total cumulative contribution rate is above 90% are retained.

At last, the reduced principal components are fused using a GMM-based method [23]. The GMM is trained by the values of the reduced principal components under the normal state. Then, the trained GMM is used to online fuse degradation features. For each new captured sample, GMM will generate $p(x_{i}|\Phi )$ using Equation (6), and the SNLLP health indicator is calculated as follows:

(15) $$SNLLP=-\log p\left(x_{i}|\Phi \right).$$

### 3.3. Incipient Fault Detection

In this module, based on the constructed SNLLP health indicator, a new alarm trigger mechanism is designed to detect the incipient fault based on the analysis of the fault typical behavior. For example, more abnormal fluctuation would appear in the signals and the amplitude of signals may increase gradually. The proposed alarm trigger mechanism is composed of two criteria: (1) in a sliding time window, the number of indicator values above the adaptive threshold has exceeded a safety number; and (2) the indicator values in the sliding time-window present an increasing trend, which is evaluated by the Spearman coefficient.

At first, the adaptive threshold based on the Chebyshev’s inequality is established to identify outliers. For an SNLLP indicator $I_{m}$, the mean and Std of its index set at time m are ${\bar{X}}_{m}$ and $S_{m}$ respectively. The Chebyshev’s inequality can be expressed as:

(16) $$P\left(\left|I_{m}-{\bar{X}}_{m}\right|\geq kS_{m}\right)\leq \frac{1}{k^{2}},\forall k>1,$$

where k is threshold tolerance. For a given confidence level $1/k^{2}$, the bound interval is expressed as $\left[{\bar{X}}_{m}-kS_{m},{\bar{X}}_{m}+kS_{m}\right]$. Equation (16) means that the probability of $I_{m}\notin \left[{\bar{X}}_{m}-kS_{m},{\bar{X}}_{m}+kS_{m}\right]$ is less than $1/k^{2}$ [24]. The former partial SNLLP indicators $\left(I_{1},I_{2},\ldots ,I_{m}\right)$ are selected to calculate the initial bound:

(17) $$T_{m}={\bar{X}}_{m}\pm kS_{m}.$$

As a new sample at time m+1 is captured, judge whether the SNLLP value of the sample belongs to the bound interval or not. If the sample belongs to the bound interval, it is added into the index set, i.e., $\left(I_{1},\ldots ,I_{m},I_{m+1}\right)$, the bound $T_{m+1}$ is recalculated:

(18) $$T_{m+1}={\bar{X}}_{m+1}\pm kS_{m+1},$$

where ${\bar{X}}_{m+1}$ and $S_{m+1}$ are the mean and standard deviation of the index set $\left(I_{1},\ldots ,I_{m},I_{m+1}\right)$. Otherwise, the sample is regarded as an outlier and the bound $T_{m+1}$ is not updated.

Since the SNLLP indicator values always perform the gradually increasing trend during the whole life cycle of bearings, the upper bound is selected as the detection threshold. Compared with random fluctuation, more abnormal fluctuation would appear when incipient faults occur. Therefore, the first criterion is defined as follows: the number of indicator values above the adaptive threshold in the sliding time-window has exceeded a safety number. The safety number is determined by the threshold tolerance and window length, which can be calculated by the following equation:

(19) $$S_{n}=\frac{l}{k^{2}},$$

where $S_{n}$ is the safety number and l is the window length. 

When incipient faults occur, the indicator always performs the gradually increasing trend. The Spearman coefficient between the indicator and time is an effective tool to evaluate the trend [25]. First, indicator ${\left\{F_{i}\right\}}_{i=1:N}$ and time ${\left\{T_{i}\right\}}_{i=1:N}$ are converted to their ranks ${\left\{f_{i}\right\}}_{i=1:N}$ and ${\left\{t_{i}\right\}}_{i=1:N}$, and then the Spearman coefficient ρ between ${\left\{f_{i}\right\}}_{i=1:N}$ and ${\left\{t_{i}\right\}}_{i=1:N}$ is computed using the following equality:

(20) $$\rho =\frac{\sum_{i=1}^{N}\left(f_{i}-\bar{f}\right)\left(t_{i}-\bar{t}\right)}{\sqrt{\sum_{i=1}^{N}{\left(f_{i}-\bar{f}\right)}^{2}\sum_{i=1}^{N}{\left(t_{i}-\bar{t}\right)}^{2}}},$$

where $\bar{f}$ and $\bar{t}$ are the means of ${\left\{f_{i}\right\}}_{i=1:N}$ and ${\left\{t_{i}\right\}}_{i=1:N}$, respectively. When ${\left\{F_{i}\right\}}_{i=1:N}$ is a monotonous increasing or decreasing function of ${\left\{T_{i}\right\}}_{i=1:N}$, the Spearman coefficient ρ equals +1 or −1. On the contrary, if ${\left\{F_{i}\right\}}_{i=1:N}$ does not have a good monotonic relation with ${\left\{T_{i}\right\}}_{i=1:N}$, the ρ will be close to 0.

Since the gradually increasing trend just performs after the incipient fault occurs, it is not a wise choice using the Spearman coefficient in all of the historical measurements. Application of the Spearman coefficient in the sliding time-window is able to increase the sensitivity of detecting the local increasing trends. Therefore, the second criterion can be defined as follows: the Spearman coefficient ρ in the sliding time-window is higher than 1/2. When the two criteria above are satisfied simultaneously, it means that an incipient fault has occurred.

## 4. Industrial Data Verification

### 4.1. Introduction to the Industrial Wind Turbine

**Figure 7:**
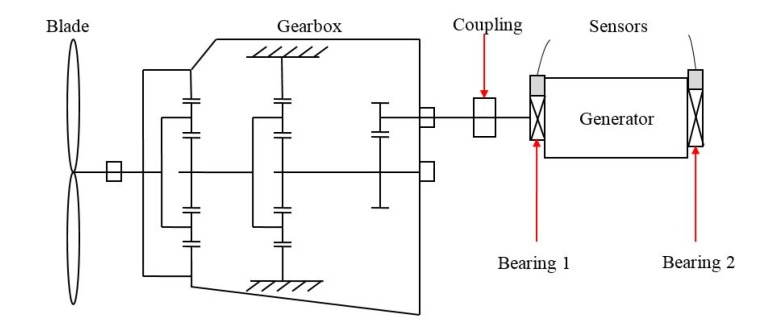
The schematic diagram of WT.

**Figure 8:**
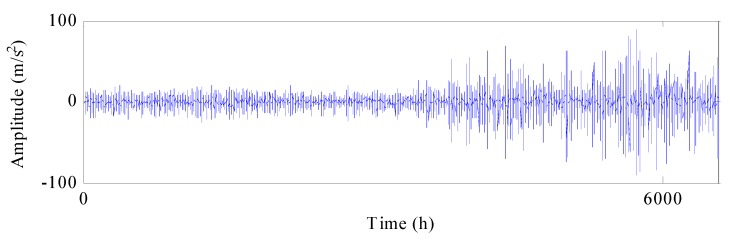
Vibration signals of bearing 2.

In this section, the proposed method is demonstrated on the incipient fault detection of generator bearings in wind turbines (WTs). As shown in Figure 7, the drivetrain of WTs mainly consists of blades, gearboxes and generators. Vibration data is acquired from condition monitoring system (CMS). The sampling frequency of accelerometers in the generator bearing is 25.6 kHz and 102,400 samples (i.e., 4 s) are recorded about each 4 h. In addition, generator speed information of WTs is collected from the supervisory control and data acquisition (SCADA) system. Due to the influence of wind speed and grid control, WT operates under varying speed characteristics. In the monitoring wind farms, from 25 September 2014 to 9 September 2015, a generator bearing appeared to naturally degenerate. Vibration signals during the whole lifetime of the bearing are shown in Figure 8.

### 4.2. RR Features Extraction of WT Bearings

Original features in Table 1 are extracted from de-noised vibration signals of the bearing by SVD, i.e., 11 time-domain features, nine frequency-domain features and 16 time-frequency-domain features. In the time-domain features, two features are extracted, which are Std of IHC and standard deviation of inverse hyperbolic sine (Std of IHS) [9]. In the frequency domain, the harmonic amplitude sum (HAS) of and the harmonic energy sum (HES) of the ball-pass frequency outer ring (BPFO), ball-pass frequency inner ring (BPFI) and ball-spin frequency (HAS-BSF) are extracted, respectively. The sum of the three HAS features and the sum of the three HES features, which are named HASS and HESS, are calculated as well. In addition, a feature named “power ratio of maximal defective frequency to mean” (PMM) [26] is used as follows:

(21) $$PMM=\frac{\max\left(EBPFO,EBPFI,EBSF\right)}{\mathrm{mean}\left(E\right)},$$

where EBPF, EBPFI, EBSF is the energy of BPFO, BPFI and BSF, mean(E) is the mean of entire frequency energy.

The 16 time-frequency-domain features are energies (E) and energy ratios (ER) of eight frequency bands decomposed by three-level wavelet packet decomposition. The db6 is selected as wavelet basis. The sampling frequency of accelerometers in the generator bearing is 25.6 kHz. Thus, the whole spectrum is divided into eight frequency bands where each frequency band has a range of 1600 Hz.

These 36 original features are classified into 22 dimensional features and 14 non-dimensional features. For the 22 dimensional features, the detailed information of the corresponding RR features extraction process is displayed as follows. The former 1000 h features and corresponding speed parameters under the normal state are used to train the ELM empirical model. Then, for a new extracted feature and speed parameter, the trained ELM empirical model is utilized to extract the RR online.

**Figure 9:**
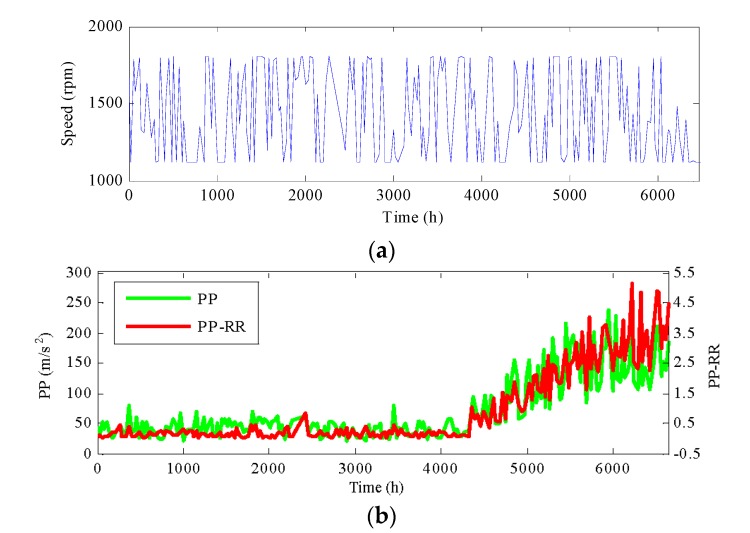
The comparison result: (**a**) the generator speed during whole life cycle; (**b**) PP and PP-RR.

In order to evaluate the performance of the proposed RR features, partial features have been intuitively compared with the corresponding RR features, which are shown in Figure 9. As mentioned before, the generator speed during the whole life cycle has larger fluctuation due to the influence of wind speed and grid control. It is seen that, with the generator speed variation in a wide range, peak-peak (PP) values have a larger and regular fluctuation. Especially when the generator speed gradually decreases, the PP values also have a decreasing trend around 6000 h. It cannot effectively reflect the bearing degradation trend at this moment. In contrast, the PP-RR has a small fluctuation with the change of speed. The amplitudes of PP-RR are stable before 4000 h. It does not have an obvious decreasing trend with the speed decreasing around 6000 h. The PP-RR presents an increasing trend during the whole life cycle, which can better reflect the degradation trend of the bearing.

### 4.3. SNLLP Health Indicator Construction of WT Bearings

**Figure 10:**
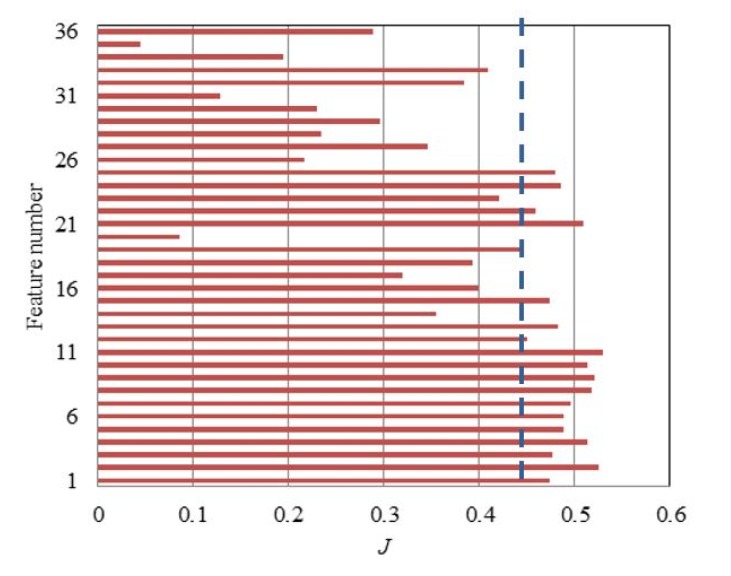
Feature selection results of bearings.

When the feature set composed of RR features and non-dimensional features is obtained, the metric in Equation (10) is used to select the features that have a good degradation performance. Generally, the features demonstrating monotonic trends are more desired. Thus, the monotonic metric is assigned higher weights than the other two metrics, which are chosen as $w_{1}=0.2,w_{2}=0.5,w_{3}=0.3$, respectively [21]. The evaluation score *J* of the 36 features are calculated and shown in Figure 10. The first half of features that have larger *J* values is selected.

**Figure 11:**
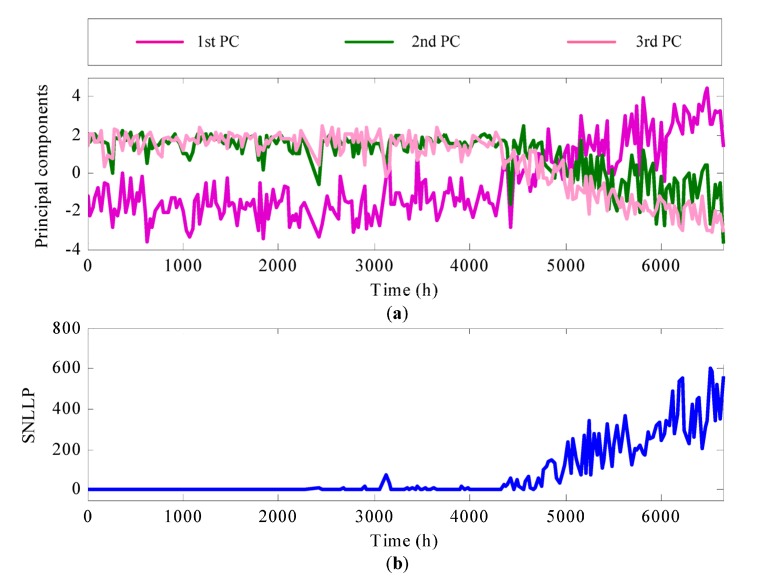
(**a**) the dimensionality reduction result by OLPP; (**b**) SNLLP health indicator.

Then, OLPP is utilized to remove redundant information in the selected 18 degradation features. The selected 18 degradation features are reduced as three principal components (PCs). The dimensionality reduction result is shown in Figure 11a. At last, the GMM-based method is utilized to fuse the three principal components. The normal data set, i.e., the former 1000 h principal components is used to construct the GMM model. The fusion result is shown in Figure 11b. It is seen that, with the fusion of these PCs, SNLLP shows more obvious degradation trends than these PCs and describes the degradation process of the generator bearing during the whole life cycle comprehensively.

### 4.4. Incipient Fault Detection of WT Bearings

Based on the statistic characteristics of the SNLLP values, the incipient faults are detected by the proposed alarm trigger mechanism in Section 3.3. The former 1000 h SNLLP values are used to calculate the initial threshold.

**Figure 12:**
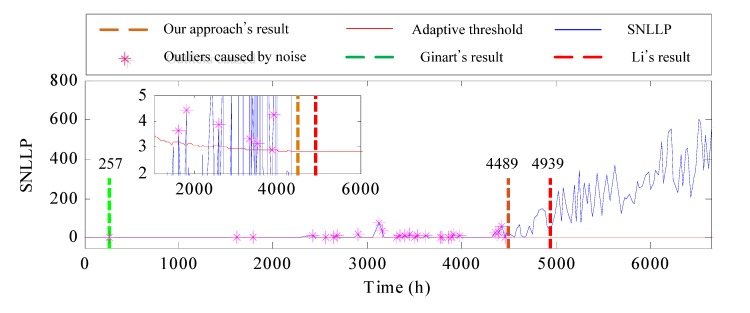
The detection result of WT incipient fault.

To demonstrate the superiority of the proposed method, another two approaches proposed by Ginart et al. [12] and Li et al. [7], respectively, are applied for comparison. The results of these approaches are shown in Figure 12. It is seen that as a new measured sample is captured, the adaptive threshold in our approach is updated continuously. There are some outliers caused by noise locating out of the adaptive threshold, which are marked by “*”. Ginart’s approach gives a false alarm during the normal state of the bearing. It is unable to exclude outliers caused by noise disturbance due to lacking a rational alarm trigger mechanism. Li’s result lags behind our approach seriously because this approach is based on all historical data. When the incipient fault appears, the change of probability is slow. However, our proposed alarm trigger mechanism has detected the incipient fault occurrence in time.

## 5. Conclusions

Aiming at the health monitoring of bearings under varying speed conditions, this paper proposes an accurate incipient fault detection method. Based on the dependence relation analysis between speed and dimensional features, RR features based on ELM are proposed to better reflect the degradation trend of bearings under varying speed conditions. Then, the SNLLP health indicator is constructed to fuse multi-parameter features. Finally, based on the constructed SNLLP health indicator, an incipient fault alarm is triggered by the proposed alarm trigger mechanism. The proposed method is demonstrated using vibration signals from test rigs of bearings and industrial WTs. The results show that the RR features are insensitive to the varying speed conditions and are able to quantitatively reflect the degradation trend of bearings. The proposed alarm trigger mechanism is more reliable and accurate for the detection of incipient faults compared with two other alarm trigger mechanisms.

Although this study has improved the accuracy of the incipient fault detection for bearings under varying speed conditions, the selection number of original features is still set subjectively. How to determine the selection number of original features adaptively is still a problem that needs to be researched in our future.

## Figures and Tables

**Table 1:** Original features extracted from the de-noised vibration signals.

Type	Time-Domain	Frequency-Domain	Time-Frequency-Domain
Dimensional indicator	F1: Standard deviation	F12: HAS-BPFO	F21–F28: E of eight bands
	F2: Peak-Peak	F13: HAS-BPFI	-
	F3: Mean-absolute	F14: HAS-BSF	-
	F4: Root mean square	F15: HASS	-
	F5: Std of IHC	F16: HES-BPFO	-
	F6: Std of IHS	F17: HES-BPFI	-
	-	F18: HES-BSF	-
	-	F19: HESS	-
Non-dimensional indicator	F7: Shape factor	F20: PMM	F29–F36: ER of eight bands
	F8: Crest factor	-	-
	F9: Impulse factor	-	-
	F10: Skewness	-	-
	F11: Kurtosis	-	-
